# Mother-child dyadic influences of affect on everyday movement behaviors: evidence from an ecological momentary assessment study

**DOI:** 10.1186/s12966-020-00951-6

**Published:** 2020-05-11

**Authors:** Chih-Hsiang Yang, Jimi Huh, Tyler B. Mason, Britni R. Belcher, Martina Kanning, Genevieve F. Dunton

**Affiliations:** 1 Department of Exercise Science, Arnold School of Public Health, University of South Carolina, 921 Assembly Street, Columbia, SC 29205 USA; 2 Department of Preventive Medicine, Keck School of Medicine, University of Southern California, Columbia, USA; 3 Department of Sports Sciences, Social and Health Sciences, University of Konstanz, Konstanz, Germany

**Keywords:** Actor effect, Partner effect, Dyads, Affective determinants, Dyadic analysis

## Abstract

**Background:**

Research has shown that affect is associated with everyday movement behaviors in children and adults. However, limited work to date has investigated dyadic influences of momentary affect on moderate-to-vigorous physical activity (MVPA) and sedentary time among children and their mothers using ecological momentary assessment (EMA).

**Methods:**

Mothers and their children (eight to 12-years-old at baseline) from the Los Angeles metropolitan area participated in a longitudinal study with six semi-annual measurement waves across three years. During each measurement wave, mothers and children reported momentary negative and positive affect via a custom smartphone-based EMA application across seven days (randomly sampled up to eight times per day). Each dyad member’s momentary affective states were used to predict their own and the other dyad member’s accelerometer-measured MVPA and sedentary time in the prompt-matched 45-min time window. Multilevel modeling within the actor-partner interdependence model (APIM) framework was applied to accommodate the nested dyadic nature of the data.

**Results:**

At the within-subject level, when children had higher-than-usual positive affect, they engaged in greater MVPA and less sedentary time in the prompt-matched 45-min window (actor effects; *ps* < .001). When mothers experienced higher-than-usual positive affect, they engaged in more sedentary time in the same 45-min window (actor effect; *p* < .001). Children’s higher-than-usual positive affect also predicted more MVPA time of their mothers (partner effect; *p* < .05). At the between-subjects level, for mothers who reported higher average negative affect than other mothers, their children overall had less MVPA and more sedentary time (partner effects *ps* < .05).

**Conclusions:**

This study extends the literature by demonstrating that mothers’ and children’s everyday physical activity and sedentary time are not only associated with their own affective states, but also may be influenced by the affective states of each other. Our findings suggest that affective states have the potential to influence movement behaviors in mother-child dyads’ everyday lives. Affective underpinnings of physical activity and sedentary behaviors should be further studied in order to develop family-based intervention strategies to influence these behaviors.

## Introduction

The high prevalence of physical inactivity among children and adolescents has become one of the most critical public health issues in modern society. One national survey indicated adults reported that obesity and insufficient exercise were the top two childhood health concerns [1]. Indeed, population-based, longitudinal studies of children and adolescents ages nine to 15 show a sharp decline in children’s device-measured moderate-to-vigorous physical activity (MVPA) and a steady increase in sedentary time as children age. Low levels of physical activity and excessive sedentary time established during childhood often persist into adolescence and adulthood that increase the risk for chronic diseases (e.g., cardiovascular disease, diabetes) [2–4]. Thus, identifying determinants of activity levels during childhood is important to inform effective prevention and intervention strategies for these behaviors and to lower chronic disease risk in adulthood.

Affective processes and determinants of health behaviors, for example the impact of stress, emotions, incidental or anticipated affect on physical activity, have been highlighted in theoretical frameworks such as the dual-processing model, the self-regulation theory, and the self-determination theory [5–8]. Research in this area using ecological momentary assessment (EMA) also found that various momentary affective states predicted future activity levels within a few minutes to several hours [9–14]. In a review synthesizing evidence from intensive longitudinal studies, momentary positive affect but not negative affect generally predicted higher levels of everyday physical activity among non-clinical adult populations [14]. Compared to adults, relatively fewer studies have investigated the temporal association between affect and activity levels specifically in children or adolescents. One study using EMA and accelerometry found that when children (ages nine to 13 years) experienced higher states of feeling energetic and lower states of feeling tired, they had more subsequent MVPA in the 30 min following the prompts. However, there were no significant associations between momentary positive or negative affect and subsequent MVPA time [9]. Another EMA study using the same child sample as the current study (ages eight to 12 years) consistently showed that momentary positive and negative affect did not serve as precursors in predicting children’s MVPA or sedentary time either in the following 30 or 60 min [15]. These studies generally investigated whether individuals’ affect predicted their own activity levels but did not consider the simultaneous interpersonal affective influences.

The current study aimed to expand the scope beyond the individual level by investigating the interpersonal affective influence on children’s activity levels. It sought to simultaneously take into account both children’s and their mothers’ affective influences on their everyday movement behaviors, including physical activity and sedentary time, using a dyadic analytic approach. This strategy may add insight into other situational and contextual factors that explain prior mixed findings on the associations between acute affect and movement behaviors in children. Research to date has shown that mothers play a key contributing role in their children’s health behaviors over time including physical activity [16]. For example, mothers are often more likely to be responsible for taking their children to parks and venues for physical activity as well as for encouraging and modeling physical activity behavior [17]. Mothers’ own physical activity-related practices are also associated with their children’s likelihood of engaging in MVPA and sedentary behavior in daily lives [18]. These maternal supportive practices that bolster children’s physical activity may be altered by mothers’ positive and negative affective states [19]. Thus, mother’s momentary affect may play a critical role in shaping her child’s daily activity levels when they are together. On the other hand, a mother may alter her behavior in response to the child’s affective states (e.g., when children feel stressed or joyful). Thus, children’s and their mothers’ activity levels may be influenced by both their own and the other person’s (mother or child) momentary affective experiences. Yet, these dyadic influences are largely overlooked in existing physical activity research, including studies using EMA. This gap has hampered researchers from understanding interpersonal affective mechanisms underlying activity levels in mother-child dyads, which, in turn, may hinder the development of effective parenting interventions to promote active lifestyles for families. Studies are needed that can disentangle the independent effects of mothers’ and children’s affective states on daily activity behaviors by testing them using dyadic modeling.

EMA data collected from smartphones and wearable devices provided real-time data that enabled the investigation of intrapersonal psychological processes and physical activity as they unfold in daily contexts [20, 21]. Innovative to this study is the application of an actor-partner interdependence model (APIM) analytic approach with multilevel (occasions nested within subjects) dyadic data to examine the independent effects of mothers’ and children’s affective experiences on the physical activity and sedentary levels of themselves and their partners. This is important to distinguish because analyzing dyadic data using a separate model for each dyad member or using a single model with a dyad-level composite score may lead to biased estimations by not accounting for the interdependent phenomena within dyads [22].

The APIM provides a framework to examine outcomes within dyads by disentangling the actor and partner effects, while considering the interdependencies that often occur within dyads [23, 24]. By simultaneously examining both the actor and the partner effects, as well as accounting for the covariations between responses from each member of a dyad, the APIM approach yields greater capacity in reflecting both the intrapersonal and interpersonal influences on individuals’ everyday behaviors [25]. The actor effects in the current study examined the momentary association of mother’s and child’s affective states with their own activity patterns, and the partner effects examined the momentary association of mother’s and child’s affective states with their dyad member’s (mother or child) activity patterns. Based on the available literature showing that momentary positive affect is generally salient in predicting more physical activity and negative affect often links to sedentary time [14, 26], we hypothesized that (a) positive affect would predict more MVPA and less sedentary time within and between subjects (i.e., both actor and partner effects) of the dyad and (b) negative affect would lead to less MVPA and more sedentary time within and between subjects (i.e., both actor and partner effects) of the dyad for both mothers and their children.

## Methods

### Participants

Participants in the current study were from the Mother’s and Their Children’s Health (MATCH) study. The MATCH study was comprised of working mothers and their children from the greater Los Angeles metropolitan area. The overall goal of this study was to examine the within-day associations of maternal stress with children’s physical activity, dietary intake, and obesity risk using novel real-time data capture strategies. Participant recruitment started in August 2014 and the study was concluded in February 2018. A total of 185 mother-child dyads who followed the study protocol and finished at least one of the six semi-annual measurement waves were included in the current analysis. In the initial lab visit at the first wave, participants signed the informed consent and reported their date of birth, sex, race, and ethnicity. Their weights (kg) and heights (m) were assessed in duplicate by trained research staff at each wave using a digital scale. Mothers’ and children’s body mass index (BMI = kg/m^2^) scores were then calculated. Children’s BMI scores were then transformed to standardized Z scores using the SAS program and growth charts (between 0 and 20 years old) provided by the CDC. A more detailed description of the MATCH study design has been published elsewhere [19].

#### Ecological momentary assessment procedures

**Fig. 1:**
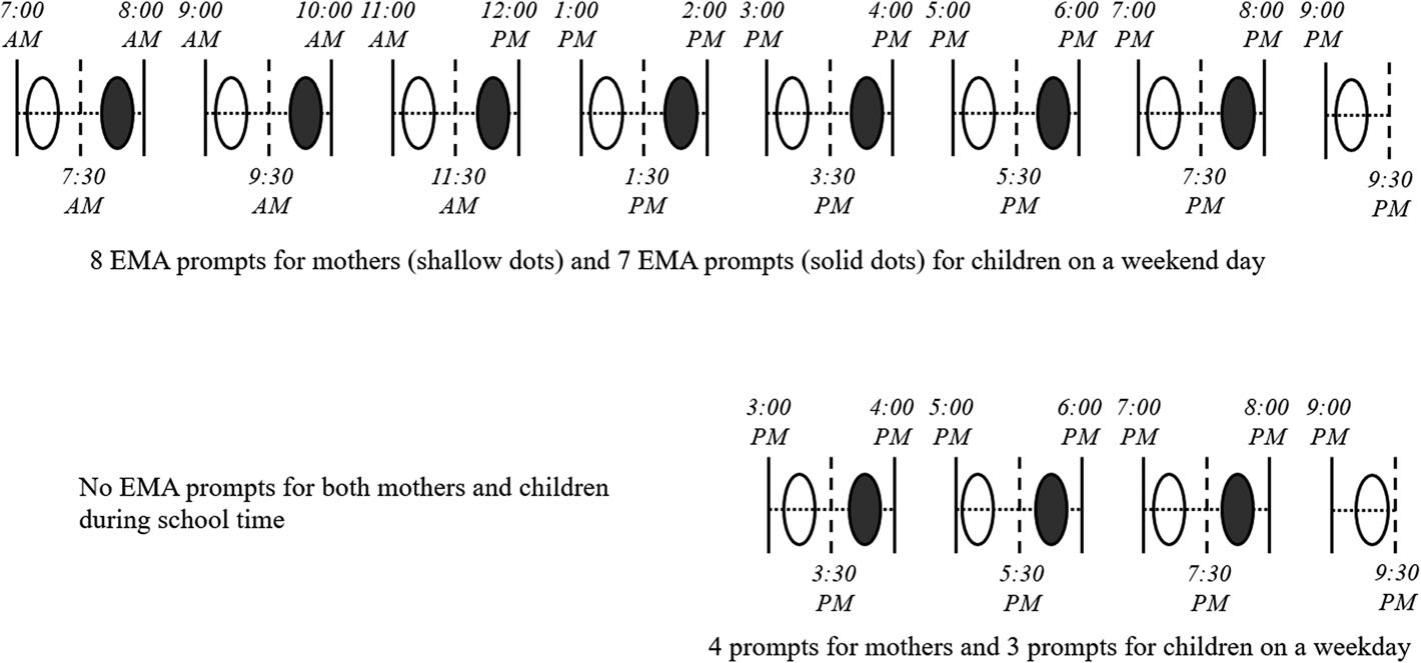
Example of the EMA sampling scheme for mothers and children applied in the current study

During each measurement wave, mother-child dyads were asked to complete a seven-day signal-contingent EMA. The EMA surveys were delivered via a customized smartphone application operated by the Android system (Google Inc., Mountainview, CA). Participants downloaded the EMA app on their own smartphone, and completed surveys were uploaded wirelessly to an internet server accessible only by study staff. Mothers and children who did not own Android smartphones were loaned a MotoG (Motorola, USA) smartphone and were instructed to connect the phone to their home wireless Internet. The time required to complete each prompted EMA survey was approximately two to three minutes, and there was a minimum one-hour interval between the random prompts. If the participants did not respond to the scheduled EMA survey, follow-up reminder signals were delivered three minutes and six minutes after the initial prompt. If there was no response after 10 min, the EMA survey became inaccessible and was treated as missing data. Mothers received eight random prompts on a weekend day and four prompts on a weekday; children received seven random prompts on a weekend day and three prompts on a weekday. To prevent potential contamination effects from answering EMA items at the same time, mothers were randomly prompted during the first half of the hour window and children were randomly prompted during the second half of the same hour (see Fig. 1).

### Measures

#### Positive and negative affect

Momentary negative affect and positive affect were assessed in each EMA survey. Two items were used to measure mothers’ positive affect (“Right before the phone went off, how (1) happy, (2) calm/relaxed were you feeling?”) and children’s positive affect (“Right before the phone went off, how (1) happy, (2) joyful were you feeling?”), respectively. Three items were used to measure mothers’ negative affect (“Right before the phone went off, how (1) frustrated/angry, (2) sad/depressed, (3) stressed were you feeling?”) and children’s negative affect (“Right before the phone went off, how (1) mad, (2) sad, (3) stressed were you feeling?”), respectively. The EMA affect questions were adapted from validated positive and negative affect schedule for children and parent [27].

Participants responded to each positive and negative affect item on a scale ranging from 1 (*not at all*) to 4 (*extremely*). Scores from the two positive affect items were averaged to represent composite indices for mothers’ and children’s positive affect, respectively. The internal consistency (ω) of the two positive affect items was high for both mothers (ω = .71) and children (ω = .90). Scores from the three items of negative affect were also averaged to represent mothers’ and children’s negative affect, respectively. The three negative items were internally consistent within both mothers (ω = 0.83) and children (ω = 0.85) in the study sample [28].

#### Physical activity and sedentary time

Mothers’ and children’s physical activity and sedentary time were measured using waist-worn Actigraph, Inc. model GT3X or WGT3X-BT accelerometers during all non-sleep time except bathing or swimming. Activity counts were recorded using a 30-s epoch, and the thresholds for MVPA and sedentary time were consistent with previous studies analyzing national surveillance data [29, 30]. Age-specific thresholds for children’s activity levels were also adjusted by applying the Freedson prediction equation [31, 32]. Non-wear time (> 60 continuous minutes of zero activity counts) and non-valid days (< 10 h of wear time) were removed from analyses. Only time windows that included at least 30 min (out of 45 min) of valid wear time within valid days were included in the analyses.

To account for the EMA delivering schedule that had mothers’ prompts generally preceded their child’s prompts by about 30 min (mean = 29.52 min; median = 29.55 min), a pair of 45-min prompt-matched time windows from each mother’s and child’s time-stamped accelerometry data were selected for analysis. The starting time of this 45-min prompt-matched accelerometry window was anchored at 15 min *after* mother’s EMA prompt time (equivalent to 15 min *before* the child’s prompt time), such that mother’s and child’s activity data time frame were matched up without discrepancy. Accumulated minutes of MVPA and sedentary time were derived from each of the 45-min prompt-matched time windows as the outcome variables.

#### Demographics, social, and temporal factors

Participants’ age (years), sex (male: yes, no), mean BMI (kg/m^2^), and race/ethnicity (Hispanic: yes, no) were included in the analysis as covariates. Time variables were derived from the date and time stamps recorded on the smartphone. When participants reported each EMA prompt, their response time (in 24 h), day of the week (weekends/weekdays), and the time difference (in minutes) between mothers’ and child’s EMA prompt for the same scheduled window were calculated. These basic demographic and temporal factors were included in the analysis as covariates based on their potential impact on daily physical activity and sedentary behavior patterns [30, 33–35]. Children also reported their social context using a multiple selection question during each EMA prompt (“Who were you with just before the phone went off?”). A binary response scale was created and the value of 1 is coded if “mother” was selected (versus all other options coded as 0).

### Data preparation and analyses

To accommodate the nested dyadic nature of the data, multilevel modeling within the actor-partner interdependence model (APIM) framework was applied for data analyses [36, 37]. Mothers’ and children’s momentary positive and negative affect EMA scores were group-mean centered (raw scores centered at each individual’s mean score) to create the mean score and deviation scores for each individual. The person-mean scores represent how individuals differ from each other (between-subject differences), and the deviations scores represent the changes from one’s own mean score (within-subject differences) at any given prompt [38, 39].

The dependent variable, MVPA minutes, was positively skewed (skewness = 4.78). Therefore, the Box-Cox method was applied to transform the distribution (optimal λ = 0.06; transformed skewness = 0.07) [40, 41]. Sedentary minutes were not severely skewed (skewness = − 0.45); thus, the original unit (minute) was retained for analyses. Multilevel models were estimated using R v3.2.2 [42] with the nlme package [43]. The two-intercept approach for analyzing distinguishable dyadic data was applied in current analyses, in which two dummy variables were created to evoke parameter estimation for mother’s and child’s variables [22, 23].

**Fig. 2:**
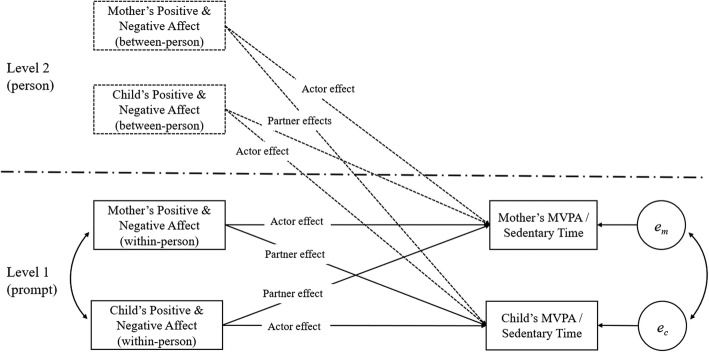
The Actor-Partner Interdependence Model analyzed within the multilevel model framework. MVPA: moderate to vigorous physical activity

A total of 39,280 EMA self-reports from mothers or children were collected across the six measurement waves, and 27,832 self-reports had at least 30 min of valid accelerometry data out of the 45-min prompt-matched time windows. To examine the potential partner effect of affect on mother’s and child’s EMA prompt-matched activity level, analyses were conducted on occasions when the child reported (on the EMA survey) that they were with their mother during the same prompt window (number of occasions = 10,766). Missing EMA and accelerometry data from either dyad member were further excluded from the analysis. The remaining 9315 prompt occasions nested within 185 mother-child dyads were analyzed using the APIM framework. A diagram of the tested dyadic multilevel model is presented in Fig. 2.

## Results

### Descriptive statistics

Mothers in the current study sample (N = 185) were predominantly Hispanic (51%), White (43%), and had a mean age of 41.03 years (*SD* = 5.86) at baseline. Children (N = 185) in the study (53% female) were mostly Hispanic (59%), White (45%), and had a mean age of 9.51 (*SD* = 0.90) at baseline. Children’s mean BMI-z score was 0.49 (*SD* = 1.10), and mothers’ mean BMI was 28.96 (*SD* = 6.58) across the six measurement waves. Forty two mother-child dyads provided valid data for each of the six measurement waves and 128 dyads (69%) provided valid data from at least four measurement waves (Median = 4, Mean = 4.04 per dyad). Across all EMA reports, each wave contributed a proportion of data ranging from 13.8% (wave 3) to 23.7% (wave 1). Approximately half (48.6%) of the EMA reports were collected on a weekday, and over three-fourths (76.8%) of the EMA reports were collected between 3 pm and 8 pm. On average, each mother-child dyad provided 28.33 (*SD* = 20.37) prompt-matched EMA reports. The averaged EMA compliance rate (responded prompts over all received prompts) was 80.3% for mothers and 76.9% for children across the six waves. Bivariate correlations between EMA compliance rates and study variables (mother or child, age, child’s gender, child’s BMI-z, response time, mother’s and children’s MVPA and sedentary time) were weak (*r*s ranged from − 0.07 to 0.12), indicating that EMA compliance was not systematically associated with key variables.

Table 1 shows descriptive statistics, correlations, and intraclass correlation coefficients (ICCs) for the main study variables using the raw scores. Both mothers and children reported low mean negative affect and moderate mean positive affect. On average, participants were being sedentary most of the time and only had a few minutes of MVPA within the 45-min prompt-matched windows. ICCs indicated that variances in negative affect, positive affect, and MVPA minutes for both mothers and children were predominately due to within-subject differences, with a relatively small proportion of variance was due to between-subject differences (ICCs ranged from 0.11 to 0.29). Approximately half of the variance in mothers’ and children’s sedentary time was due to within-subject differences.

**Table 1:** Descriptive statistics, correlations, and intraclass correlation coefficients of the main variables in the analysis (N of dyads = 185)

	Mean	*SD*	Median	Range	Negative affect	Positive affect	MVPA mins	Sedentary mins
***Mothers***								
Negative affect	1.381	0.532	1	4	(0.214)	−0.528**	*− 0.066*	*0.055*
Positive affect	2.636	0.745	3	4	−0.568**	(0.134)	*0.026*	*0.026*
MVPA mins	1.177	3.057	0	45	0.011	−0.014	(0.129)	−0.591**
Sedentary mins	29.855	9.016	31	45	−0.021	0.056**	−0.444**	(0.529)
***Children***								
Negative affect	1.260	0.515	1	4	(0.293)	−0.278**	*−0.036*	*0.027*
Positive affect	3.032	0.944	3	4	−0.344**	(0.111)	*0.131*	*−0.139*
MVPA mins	2.345	4.125	1	45	−0.020	0.042**	(0.235)	−0.645**
Sedentary mins	26.847	9.310	27	45	0.016	−0.055**	− 0.600**	(0.481)

*Note.* Intraclass correlation coefficients (ICCs) were calculated in the diagonal parentheses of the matrix. The between-subject correlations are above the diagonal and italicized and the pooled within-subject correlations are below the diagonal (e.g., the between-subject corration for mothers’ positive and negative affect was − 0.53). The EMA response scale for negative and positive affect ranged from 1 to 4. MVPA and Sedentary time ranged from 1 to 45 min

*p < .05 **p < .01

### Demographic and time variables related to MVPA and sedentary time

As shown in Tables 2 and 3, less MVPA and more sedentary time were observed later in the day for both mothers and children. Compared to girls, boys had more MVPA, but there were no sex differences in sedentary time (*p* = .18). Children’s age at each wave was associated with less MVPA and more sedentary time. Mothers who were relatively younger at baseline had more sedentary time. Children’s mean BMI-z score across waves was negatively related to MVPA but not sedentary time (*p* = .87). Children and mothers engaged in less MVPA on weekend days compared to weekdays during the EMA prompt-matched time windows. These findings were controlled for all other covariates in the model.

**Table 2:** Dyadic multilevel model predicting mothers’ and children’s prompt matched MVPA (N of dyads = 185; N of observations = 9315)

Model	Estimate	Std. Error
**Fixed Effects**		
Mother – intercept	0.850**	0.264
Child - intercept	3.139***	0.383
Mother - EMA response time	−0.050***	0.008
Child - EMA response time	−0.039***	0.010
Child - male (vs female)	0.095*	0.039
Mother - age at each wave	0.015	0.010
Child - age at each wave	−0.127***	0.013
Mother - baseline age	−0.011	0.025
Child - baseline age	−0.012	0.010
Mother - Hispanic (vs non-Hispanic)	−0.012	0.037
Child - Hispanic (vs non-Hispanic)	−0.054	0.043
Child - mean BMI	−0.012**	0.005
Mother - EMA prompt time difference	−0.001	0.001
Child - EMA prompt time difference	0.001	0.001
Mother - weekend (vs weekday)	−0.064**	0.021
Child - weekend (vs weekday)	−0.082**	0.026
***Actor Effects***		
Mother - within-subject positive affect	−0.026	0.018
Mother - between-subject positive affect	0.026	0.050
Child - within-subject positive affect	0.071***	0.016
Child - between-subject positive affect	−0.026	0.040
Mother - within-subject negative affect	0.010	0.025
Mother - between-subject negative affect	−0.011	0.074
Child - within-subject negative affect	0.029	0.027
Child - between-subject negative affect	0.016	0.087
***Partner Effects***		
Child - within-subject positive affect on mother	0.030*	0.013
Child - between-subject positive affect on mother	−0.058	0.033
Mother - within-subject positive affect on child	0.014	0.023
Mother - between-subject positive affect on child	−0.038	0.059
Child - within-subject negative affect on mother	0.009	0.022
Child - between-subject negative affect on mother	−0.084	0.073
Mother - within-subject negative affect on child	−0.004	0.030
Mother - between-subject negative affect on child	−0.201*	0.088
**Random Effects**		
Mother - intercept standard deviation	0.181	
Child - intercept standard deviation	0.206	
Correlation between random intercepts	0.546	
Mother - residual standard deviation	0.612	
Child - residual standard deviation	0.758	
Correlation between residuals	0.060	

*p < .05 **p < .01 ***p < .001. MVPA minutes are log-transformed

**Table 3:** Dyadic multilevel model predicting mothers’ and children’s prompt matched sedentary behavior (N of dyads = 185; N of observations = 9315)

Model	Estimate	Std. Error
**Fixed Effects**		
Mother - intercept	28.327***	4.072
Child - intercept	5.780	4.651
Mother - EMA response time	0.546***	0.104
Child - EMA response time	0.392***	0.108
Child - male (vs female)	−0.631	0.475
Mother - age at each wave	0.242	0.141
Child - age at each wave	1.089***	0.147
Mother - baseline age	0.278	0.303
Child - baseline age	−0.349*	0.148
Mother - Hispanic (vs non-Hispanic)	−0.423	0.579
Child - Hispanic (vs non-Hispanic)	0.087	0.505
Child - mean BMI	0.009	0.057
Mother - EMA prompt time difference	0.008	0.010
Child - EMA prompt time difference	−0.002	0.010
Mother - weekend (vs weekday)	−0.068	0.288
Child - weekend (vs weekday)	0.018	0.300
***Actor Effects***		
Mother - within-subject positive affect	1.122***	0.254
Mother - between-subject positive affect	0.122	0.774
Child - within-subject positive affect	−0.640***	0.189
Child - between-subject positive affect	0.083	0.470
Mother - within-subject negative affect	0.316	0.339
Mother - between-subject negative affect	0.326	1.138
Child - within-subject negative affect	0.214	0.311
Child - between-subject negative affect	−0.537	1.007
***Partner Effects***		
Child - within-subject positive affect on mother	−0.188	0.182
Child - between-subject positive affect on mother	0.776	0.512
Mother - within-subject positive affect on child	0.012	0.265
Mother - between-subject positive affect on child	0.762	0.698
Child - within-subject negative affect on mother	−0.360	0.299
Child - between-subject negative affect on mother	0.067	1.114
Mother - within-subject negative affect on child	0.280	0.353
Mother - between-subject negative affect on child	2.153*	1.036
**Random Effects**		
Mother - intercept standard deviation	2.976	
Child - intercept standard deviation	2.485	
Estimated correlation between random intercepts	0.441	
Mother - residual standard deviation	8.418	
Child - residual standard deviation	8.761	
Estimated correlation between residuals	0.046	

*p < .05 **p < .01 ***p < .001

### Actor and partner effects of positive and negative affect on movement behaviors

Table 2 shows results for the multilevel APIM predicting MVPA. Controlling for negative affect and other covariates, there were significant within-subject actor and partner effects. On occasions when children had higher positive affect than their typical levels, they (b = 0.07, *p* < .001) and their mothers (b = 0.03, *p* < .05) engaged in more MVPA during the EMA prompt-matched time windows. At the between-subject level, a partner effect was observed for mothers such that their mean levels of negative affect across all EMA prompts were negatively associated with children’s MVPA (b = − 0.20, *p* < .05). No significant actor effects were identified at the between-subject level, and there were no within-subject actor or partner effects for negative affect.

Table 3 shows the results of the multilevel APIM predicting sedentary time. Within-subject actor effects for positive affect were observed for both mothers and children after controlling for negative affect and other covariates. When children reported higher positive affect than their typical levels, they engaged in less sedentary time (b = − 0.64, *p* < .001). In contrast, when mothers reported higher positive affect than their typical levels, they engaged in more sedentary time within the prompt-matched time window (b = 1.12, *p* < .001). At the between-subject level, one partner effect of negative affect was observed. Among mothers who, on average, reported greater negative affect than other mothers, their children engaged in more sedentary time within the prompt-matched time window (b = 2.15, *p* < .05). There were no significant partner effects at the within-subject level, and there were no significant actor effects at the between-subject level.

Adjusting for all the actor and partner effects and covariates, the correlations of the random effects at the bottom of Tables 2 and 3 revealed the interdependency of activity levels in mother-child dyads. The positive correlation between the random intercept of mothers’ and children’s MVPA indicated that mothers who had higher average MVPA tended to have children who also had higher average MVPA (*r* = 0.55; 95%CI = 0.31–0.73). Similarly, for children who had higher average sedentary time, their mothers also tended to have higher average sedentary time (*r* = 0.44; 95%CI = 0.24–0.64). The positive covariation between mother’s and child’s activity levels were also observed at the prompt (residual) level. At any given EMA prompt, mother’s prompt-matched MVPA and sedentary time were positively related to the child’s prompt-matched MVPA (*ρ* = 0.06; 95%CI = 0.04–0.08) and sedentary time (*ρ* = 0.05; 95%CI = 0.03–0.06), respectively.

## Discussion

The current study applied the APIM framework to examine actor and partner effects of momentary affect on physical activity and sedentary time in mother-child dyads using EMA and accelerometry data. Results suggested that associations between momentary affective states and activity levels in mother-child dyads may be determined by both intra-individual and inter-personal processes. These findings support the notion that the close relationship between mothers and children is an important contextual factor that should be considered when studying human behaviors [44]. Affective processes and determinants of physical activity should be studied using an interpersonal perspective beyond the individual level. Effective intervention strategies to promote active lifestyles through affective determinants may also need to target both parents and children to optimize effectiveness.

The hypothesis that positive affect would predict more MVPA and less sedentary time was primarily supported by children‘s actor and partner effects. One recent study consistently reported a positive association between parent’s and children’s device-based activity levels during weekends and after-school periods [45]. Children with higher positive affect during off-school time may be more likely to ask their mother to engage in physical activity with them or take them to a venue for physical activity which may, in turn, provide mothers with an opportunity to increase their activity levels. Further, children’s positive affect may be higher during occasions when mothers and children engage in planned activities together that would promote MVPA. This possibility should be further tested in future studies using EMA in conjunction with self-reported physical activity or by analyzing passive sensing location-based data from Global Positioning System (GPS) [46].

Mothers’ momentary positive affect was associated with more sedentary time for themselves, which is the opposite of what was found for children. One possible reason may be that mothers often take on more responsibilities in housework and childcare compared to fathers in a general family context [47]. This finding may partially reflect mothers’ “off-duty” sedentary resting/leisure time. That is, mothers may report feelings that reflect being more relaxed or calm (as assessed by the EMA item) at times when they finished or have less housework and childcare duties, leading to more sedentary time. Future studies should distinguish among the various facets of positive and negative affect to better understand how they are related to different movement behaviors.

The hypothesis that negative affect would predict more sedentary time and less MVPA was partially supported by two between-subject partner effects. Children of mothers who had higher negative affect overall engaged in less MVPA and more sedentary time. The EMA affect items specifically asked mothers how stressed and sad they were feeling, which are closely linked to chronic stress and depression symptoms [48, 49]. Our results thus support findings from previous review articles showing that higher maternal stress and chronic depression were associated with lower levels of physical activity, higher levels of sedentary behavior, and greater obesity risk in children [50, 51]. Long-lasting maternal stress and depression may also lead to fewer mother-child interactions and passive parenting practices that indirectly impact children’s daily activity levels [52]. Further, mothers who constantly experience elevated negative affect may have already established an inactive lifestyle that influences children’s activity levels through maternal modeling [53]. Our study using intensive longitudinal design revealed similar between-subject phenomena established mostly on previous cross-sectional studies.

Compared to the past EMA studies, our results revealed different findings regarding the within-subject associations between momentary affect and movement behaviors in children. Previous EMA studies were designed to understand children’s affective states on their activity levels without considering the simultaneous effects of other people’s affective states, which could confound or suppress the effects of children’s affect on their own behaviors. The current study exclusively investigated occasions only when mother and child reported being together (and thus not alone or with people other than the mother). Thus, the observed phenomena may be context-dependent and may not be applied to any given occasion or on occasions when children are with other family members.

Positive affect may be an important intervention target to promote active lifestyles within a family context, considering its beneficial impact identified in both models. Real-time interventions may be developed to take advantage of moments of high positive affect to trigger just-in-time support for promoting physical activity or breaking up sedentary time in mothers and children [54]. Moreover, the partner effects of mothers’ overall negative affect on children’s inactivity level (i.e., less MVPA and more sedentary time) may inform the necessity of addressing mothers’ psychological health on children’s activity patterns. Stress and affect management strategies, such as mindfulness-based stress reduction skills, could be included in family-based interventions to help mothers cope with chronic adverse affective experiences that may otherwise lead to unhealthy lifestyles in their children [55].

This is the first study to investigate the actor and partner effects of momentary affective states on physical activity and sedentary time in the everyday lives of mother-child dyads. One of the strengths of this study is the intensive longitudinal study design that collected six semi-annual waves of paired EMA and accelerometry data from mother-child dyads. Another unique contribution of the current study is the application of multilevel dyadic modeling that accounts for the nested and interdependency data structure across occasions. However, some noticeable limitations may restrict generalizability of our findings. To reduce participant burden, our EMA items only assessed several facets of positive and negative affect. Other types of affect (e.g., anxiety, pride) and physical feeling states (e.g., fatigue, pain) may also be relevant in predicting activity [5]. Also, mothers in this study were employed around the metropolitan area, therefore, results may not be generalized to household mothers recruited from other geographic regions or mothers who work from home. Across the six measurement waves, children’s ages spanned from eight to 14 years old in this study (eight to 12 years old at baseline and 10–14 years at the end of the study), the findings may not be applied to children in other age ranges. The current study did not consider other family members’ (e.g., sibling(s), father) influences when children were with them. Father-child or parent-child (triadic) interpersonal influences should be considered in studying children’s activity levels within the family context in future research [17, 56]. Due to the time-varying nature of affect, there is a lack of clear guidelines in terms of the optimal time window to study the affective processes underlying everyday movement behaviors using EMA. The influence of affective states on activity may operate on shorter or longer time frames, or it may vary depending on the relative position of the time window with reference to the EMA prompt. The current EMA study is observational and we did not manipulate participants’ affect, so it is unable to infer causality between affect and everyday activities. Since the random EMA prompts could have occurred before, during, or after movement behaviors, it is possible that some EMA occasions examined how affective experiences during or after a behavior predicted continuation of that behavior (instead of initiation of it). Building upon prior research on affective precursors of physical activity in adults’ and children’s everyday settings [9–14], our study particularly investigated this direction of the affect-activity association in mothers and their children. To guide future intervention development in a family context, it will be valuable to identify the preceding events or activities (e.g., family events, interpersonal conflicts, daily hassles) that link to parent’s and children’s affective states. Unfortunately, our study design is unable to capture a variety of preceding events and pinpoint precursors that lead to specific affect in mothers and children. Future studies should create brief follow-up EMA questions by asking participants to provide potential causes of their current positive and negative affect experiences.

## Conclusion

This study applies a novel methodology using a dyadic multilevel model to analyze both EMA and device-based activity data form mother-child dyads across three years. Our findings suggest that, when mothers and their children are together, their affective states may modulate their own and their child’s daily activity levels. This study provides support for the need to develop novel ecological momentary interventions (i.e., interventions carried in real-time in individuals’ everyday setting) and to include effective parenting strategies and stress management skills in maternal- and family-based child health promotion interventions. Dyadic analytic models should be applied to future EMA and physical activity research to better control interpersonal or social influences imbedded in real-life human behaviors.

## Data Availability

The datasets used and analyzed during the current study are available from the corresponding author on reasonable request.

## Abbreviations

EMA: Ecological momentary assessment
MVPA: Moderate-to-vigorous physical activity
APIM: Actor-partner interdependence model
ICC: Intraclass correlation coefficients
BMI: Body mass index
